# Supplementary material for: De novo reconstruction of human adipose transcriptome reveals conserved lncRNAs as regulators of brown adipogenesis
Source: Nat Commun. 2018 Apr 6;9:1329. doi: 10.1038/s41467-018-03754-3 (PMC5889397; doi:10.1038/s41467-018-03754-3)
Supplement: Supplementary file 1 — Supplementary Information [file 41467_2018_3754_MOESM1_ESM.pdf]

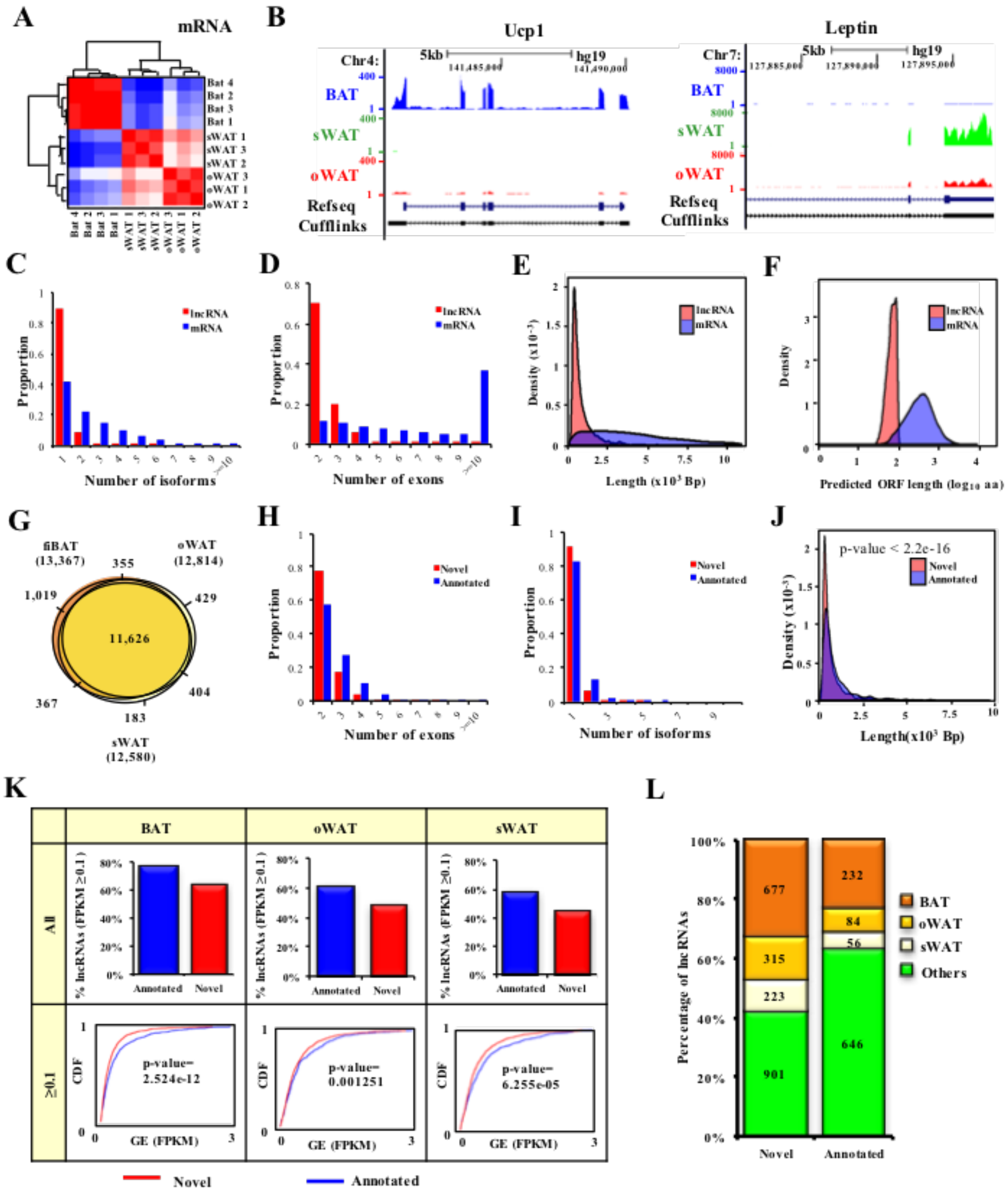

Supplementary Figure. 1 Characterization of human adipose lncRNA and comparison between novel and annotated lncRNA, related to Figure 1.

(A) Heatmap of clustered correlation between 10 human adipose RNA-seq samples based on their mRNA expression.

(B) Examples of de novo transcript structure generated by Cufflinks and their corresponding RefSeq annotations.

(C) Distribution of isoform numbers per lncRNA and mRNA.

(D) Distribution of exon numbers per lncRNA and mRNA.

(E) Transcript length distribution of lncRNA and mRNA.

(F) ORF length distribution of lncRNA and mRNA.

(G) Overlaps of detectable mRNAs (FPKM>1 for all analysed samples) among fiBAT, SUB and OME.

(H) Distribution of exon number in novel and annotated lncRNAs. Mann-Whitney U test p=1.909e-15

(I) Distribution of isoform number in novel and annotated lncRNAs. Mann-Whitney U test $p < 2.2 \times 10^{-16}$

(J) Transcript length distribution of novel lncRNAs and annotated lncRNAs. Mann-Whitney U test p-value < 2.2e-16

(K) Novel lncRNAs are significantly lower expressed than annotated lncRNAs. (Top) When FPKM ≥0.1 is used the threshold of detection, the proportion of detectable lncRNAs are consistently higher in annotated than the novel category for all 3 fat tissues. (Bottom) The cumulative fraction of novel and annotated lncRNAs according to their FPKM.

(L) Bar chart of lncRNAs uniquely expressed in fiBAT, oWAT, sWAT and in at least two fat tissues (others). A higher proportion of tissue-unique lncRNAs is found in novel than annotated group.

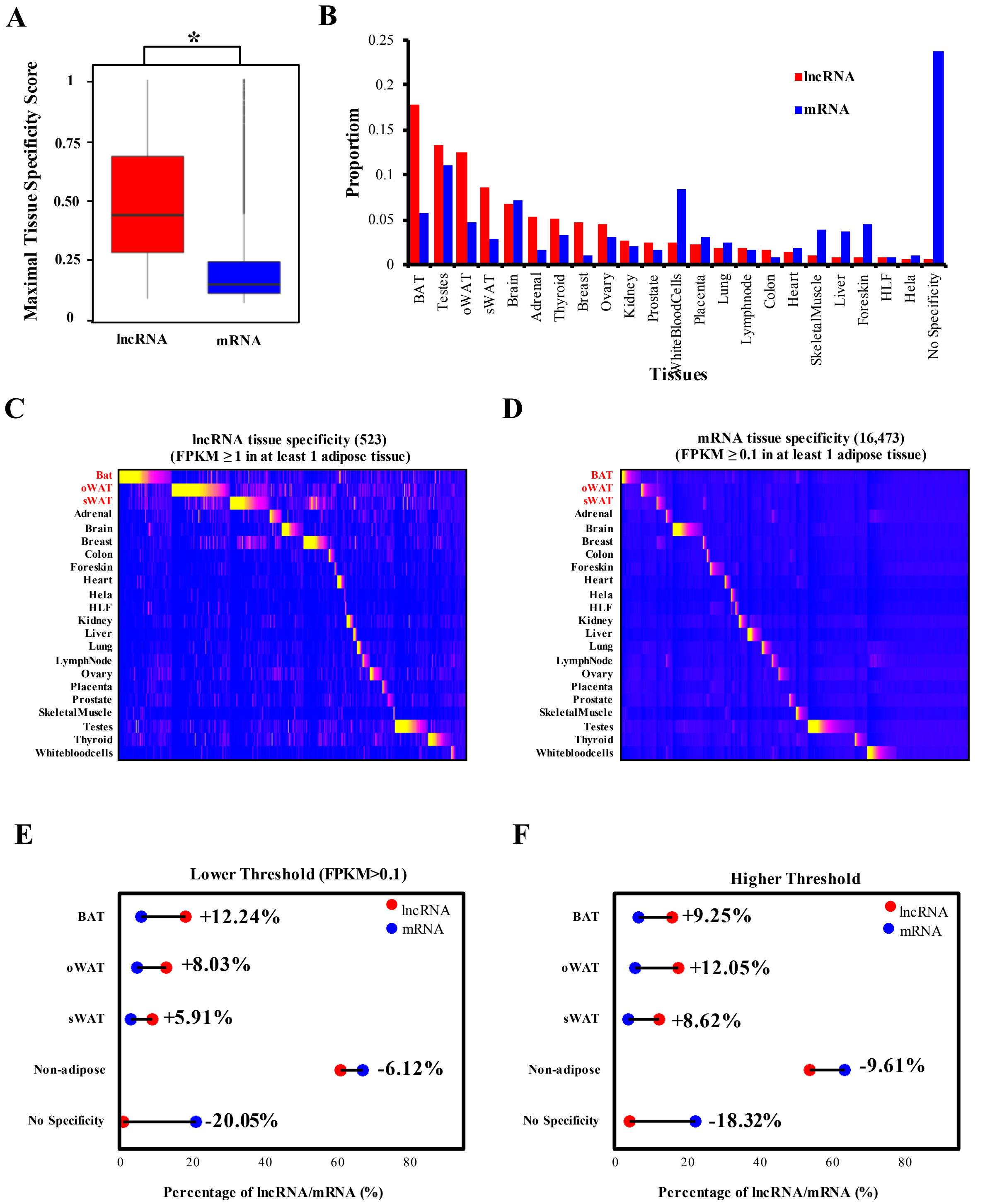

Supplementary Figure. 2 Higher tissue specificity of human adipose lncRNA than mRNA is independent of gene abundance and fractional expression thresholds, related to Figure 2.

(A) Boxplot depicting maximal specificity score distribution of lncRNAs and mRNAs. $p < 2.2x10^{-16}$

(B) Proportion of tissue specific lncRNAs or mRNA found in each of the examined tissues. 23.7% of the mRNAs do not show tissue specificity compared to a mere 0.6% of the lncRNAs.

(C) Relative abundance of 523 lncRNAs (rows) with FPKM ≥ 1 in at least one adipose tissue across 22 examined tissues (columns). Colour density represents the fractional expression of lncRNAs in each tissue relative to all examined tissues.

(D) Abundance of 16,473 adipose expressed mRNA (rows) with FPKM ≥ 0.1 in at least one adipose tissue across 22 examined tissues (columns). Colour density represents the fractional expression of mRNAs in each tissue relative to all examined tissues.

(E) Scatterplot of the percentage of lncRNA or mRNA specifically expressed in BAT, oWAT, sWAT or other non-adipose tissues using a lower expression threshold (mRNA and lncRNA FPKM≥ 0.1). Difference in percentage between lncRNA and mRNA have been indicated.

(F) Scatterplot of the percentage of lncRNA or mRNA specifically expressed in BAT, oWAT, sWAT or other non-adipose tissues using a higher expression threshold (mRNA and lncRNA FPKM≥ 1). Difference in percentage between lncRNAs and mRNAs has been indicated.

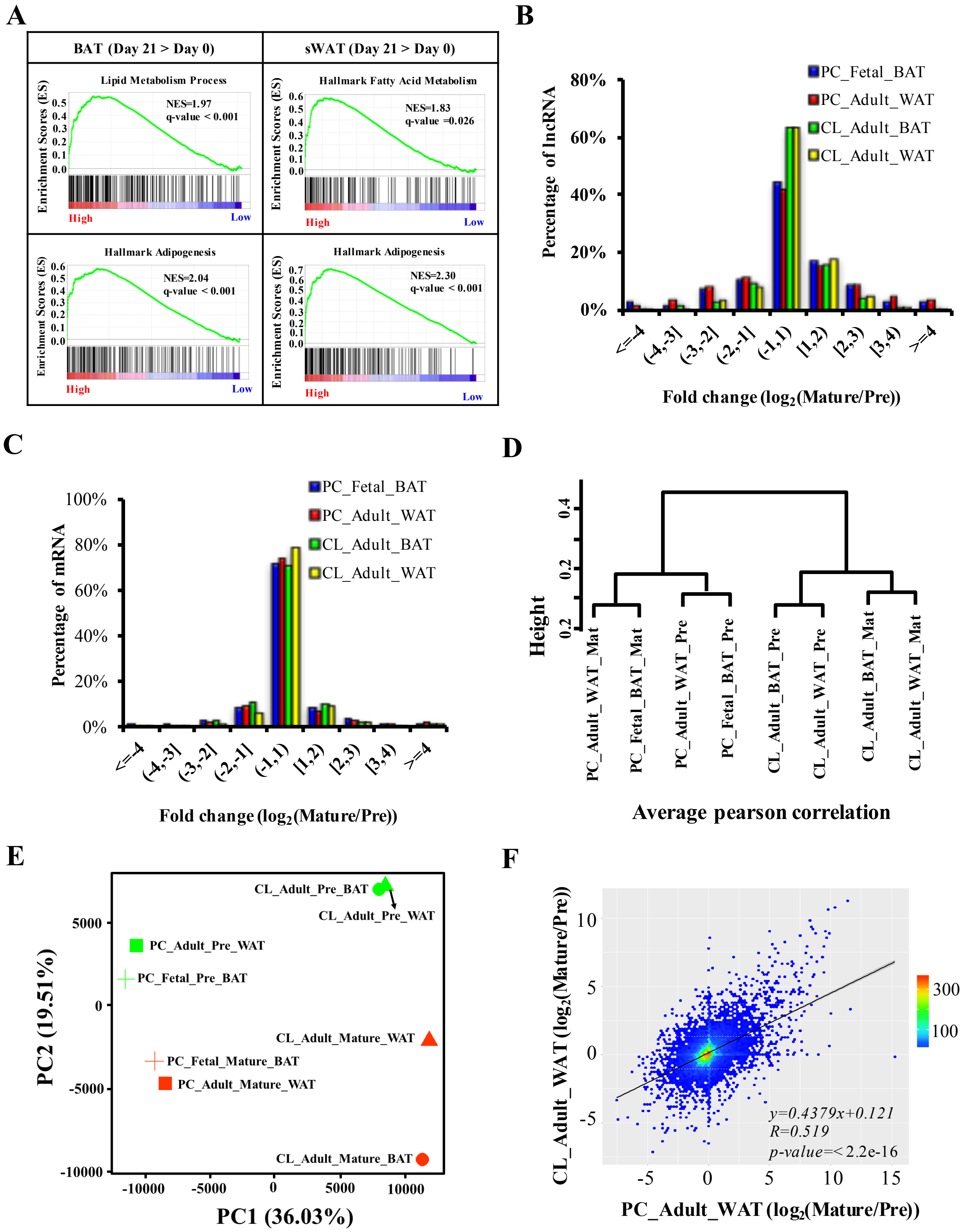

Supplementary Figure. 3 Global expression changes during adipogenesis in human primary cells and cell lines, related to Figure 3.

(A) GSEA Gene set enrichment analysis on up-regulated genes during cultured human brown and white adipogenesis using RNA-seq data (Day 0 vs Day 21).

(B-C) Distribution of expression changes during adipogenesis for mRNA (*B*) and lncRNA (*C*).

(D-E) Hierarchical clustering (*D*) and PCA (*E*) analyses of eight samples using mRNA expression profiles from RNA-seq separated primary cells from cell line.

(F) Hexbin plot of global gene expression changes for 16,274 mRNAs detectable in cultured primary (PC_Adult_WAT) and cell line during adipogenesis (CL_Adult_WAT). Density of the data points are represented by colour scales.

*CL: Cell line ; PC: Primary cells

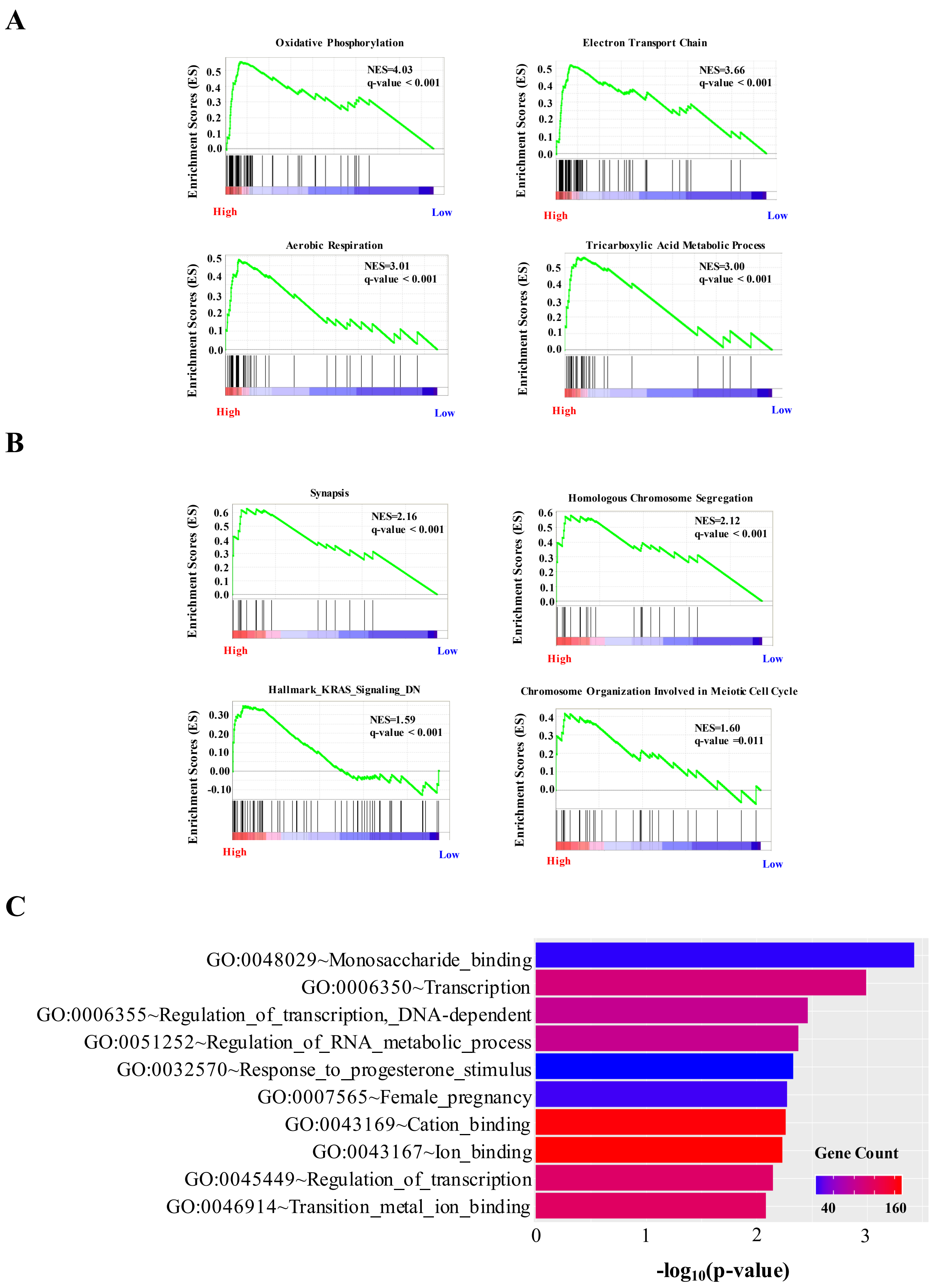

Supplementary Figure. 4 Global expression changes in human adipose tissues upon cold exposure

(A) GSEA Gene set enrichment analysis on up-regulated genes upon cold exposure in BAT.

(B) GSEA Gene set enrichment analysis on up-regulated genes upon cold exposure in WAT.

(C) Top ten enriched gene ontology processes of the up-regulated mRNAs in WAT upon cold exposure.

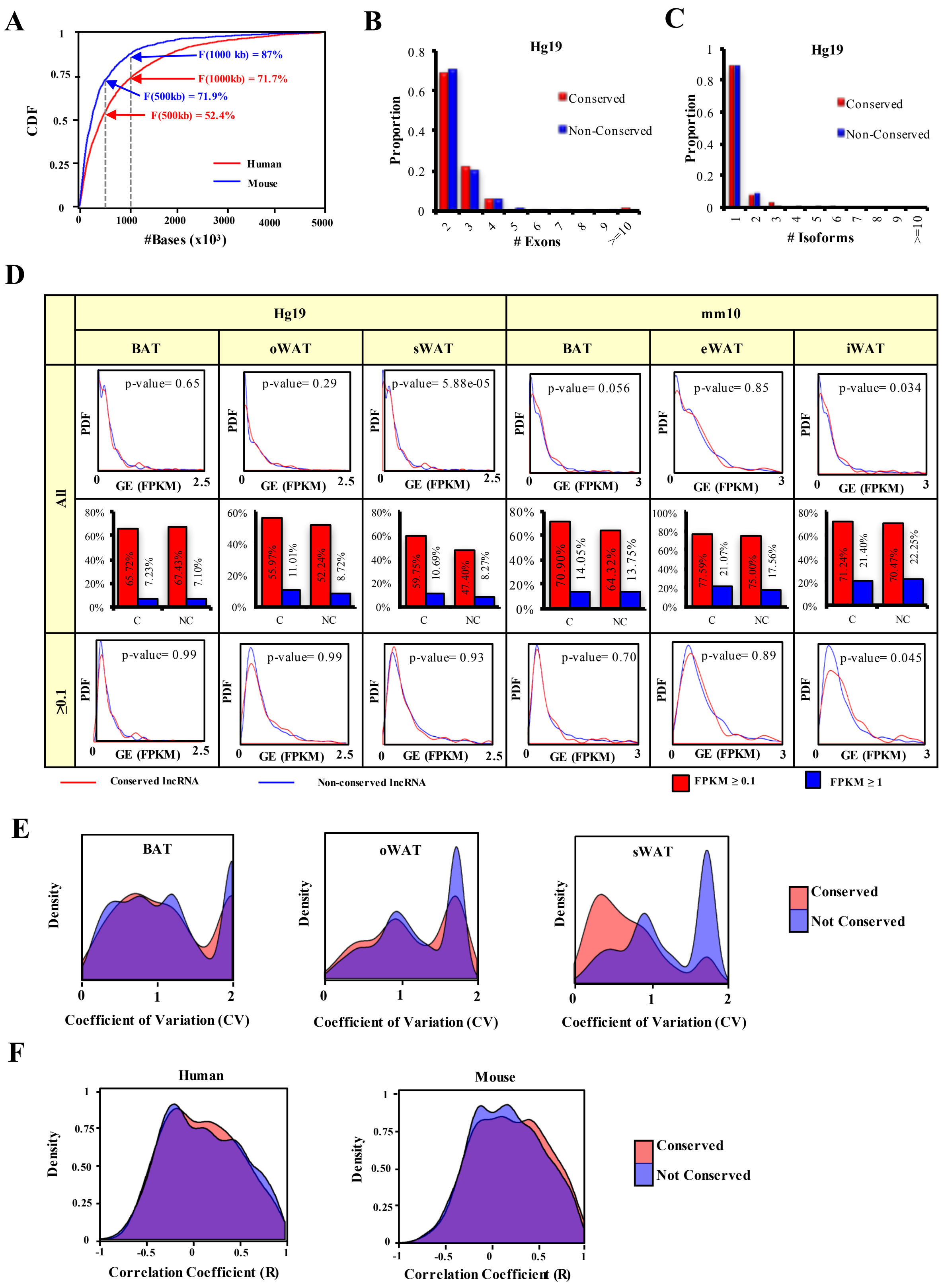

Supplementary Figure. 5 Characterization of conserved lncRNAs, related to Figure 5.

(A) Cumulative distribution of lncRNAs based on the further distance (bases) to either nearest up or downstream protein-coding gene on the same strand. Based on the *de novo* constructed catalogue,

71.9% of the mouse lncRNAs have two flanking protein-coding gene within 500 kb (same strand) compared to 52.4% in human.

(B) Distributions for exon number per transcript of novel and annotated lncRNAs.

(C) Distributions for isoform number per transcript of novel and annotated lncRNAs.

(D) Comparison of the distributions of gene expression between conserved and non-conserved lncRNA among analyzed adipose tissues in human and mouse under varying expression thresholds (Top panel: no cutoff, middle panel: lncRNAs binarized into FPKM<0.1 and FPKM ≥0.1, bottom panel: FPKM ≥0.1).

(E) Distributions for coefficient of variation (CV) in BAT, oWAT and sWAT. CV of conserved lncRNAs are significantly lower than non-conserved lncRNAs in sWAT (Mann-Whitney U test p-value < 2.2e-16), while no differences are found in BAT (Mann-Whitney U test p-value = 0.7107) or oWAT (Mann-Whitney U test p-value = 0.2339).

(F) Distributions of correlation coefficients between lncRNAs and their nearby mRNAs. No significant difference was found when conserved and non-conserved lncRNAs were compared. (Human: Mann-Whitney U test p-value = 0.7533, Mouse: Mann-Whitney U test p-value = 0.05794)

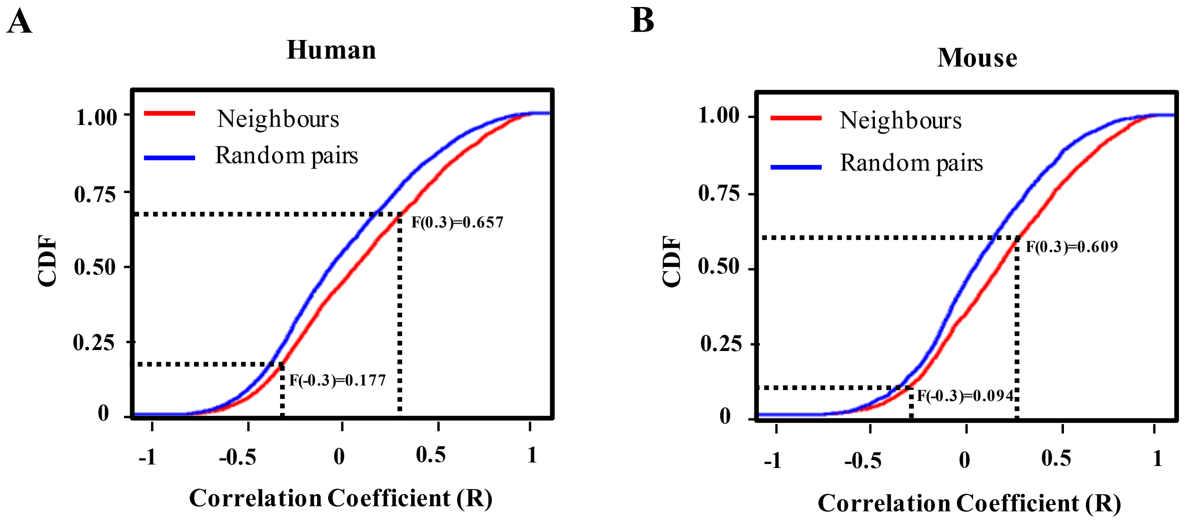

Supplementary Figure. 6 Expression correlation coefficient of lncRNA with their cis targets.

(A-B) Cumulative density functions of correlation coefficients between lncRNAs and their nearby mRNAs (red), in comparison with those between lncRNAs and random mRNAs (blue) in human (*A*) and mouse species (*B*).

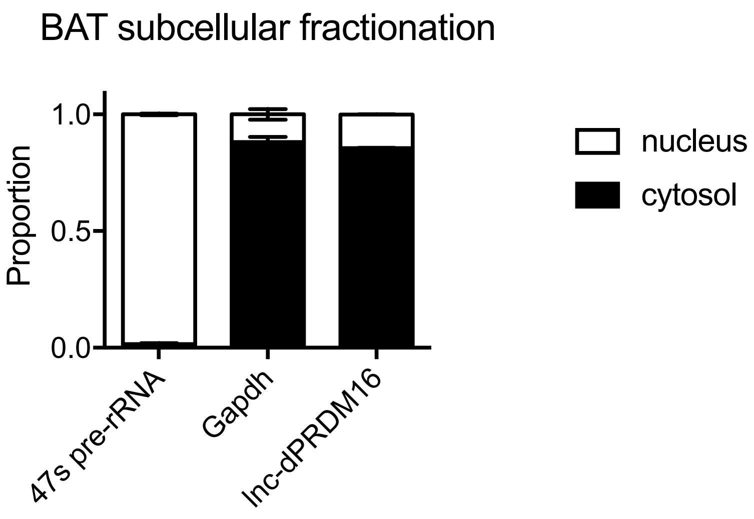

Supplementary Figure. 7 Cellular localization of lnc-dPRDM16 in BAT.

The same amount of RNA was used for realtime PCR. The relative abundance was calculated according to the CT value. n=3, error bars represent mean ± SEM.

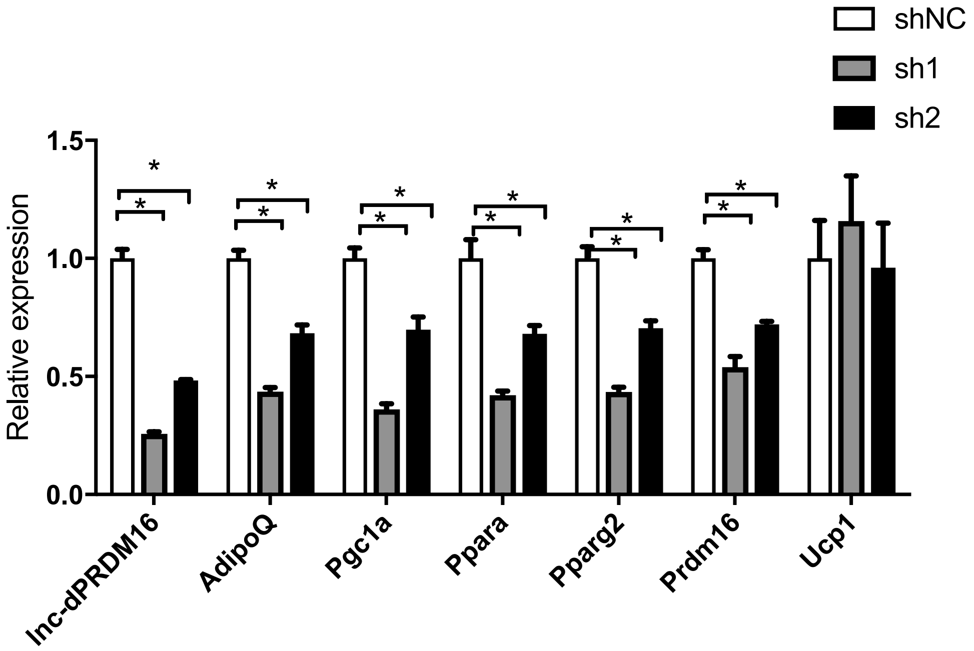

Supplementary Figure. 8. Knockdown of lnc-dPRDM16 in iWAT culture results in impaired adipogenesis.

Retirviral shRNAs were used to knock down lnc-dPRDM16 in primary white pre-adipocyte culture, followed by induction of differentiation for 5 days. Realtime PCR was performed to examine the marker expression. Error bars represent mean ± SEM, n = 4, * $P<0.05$, Student T test.

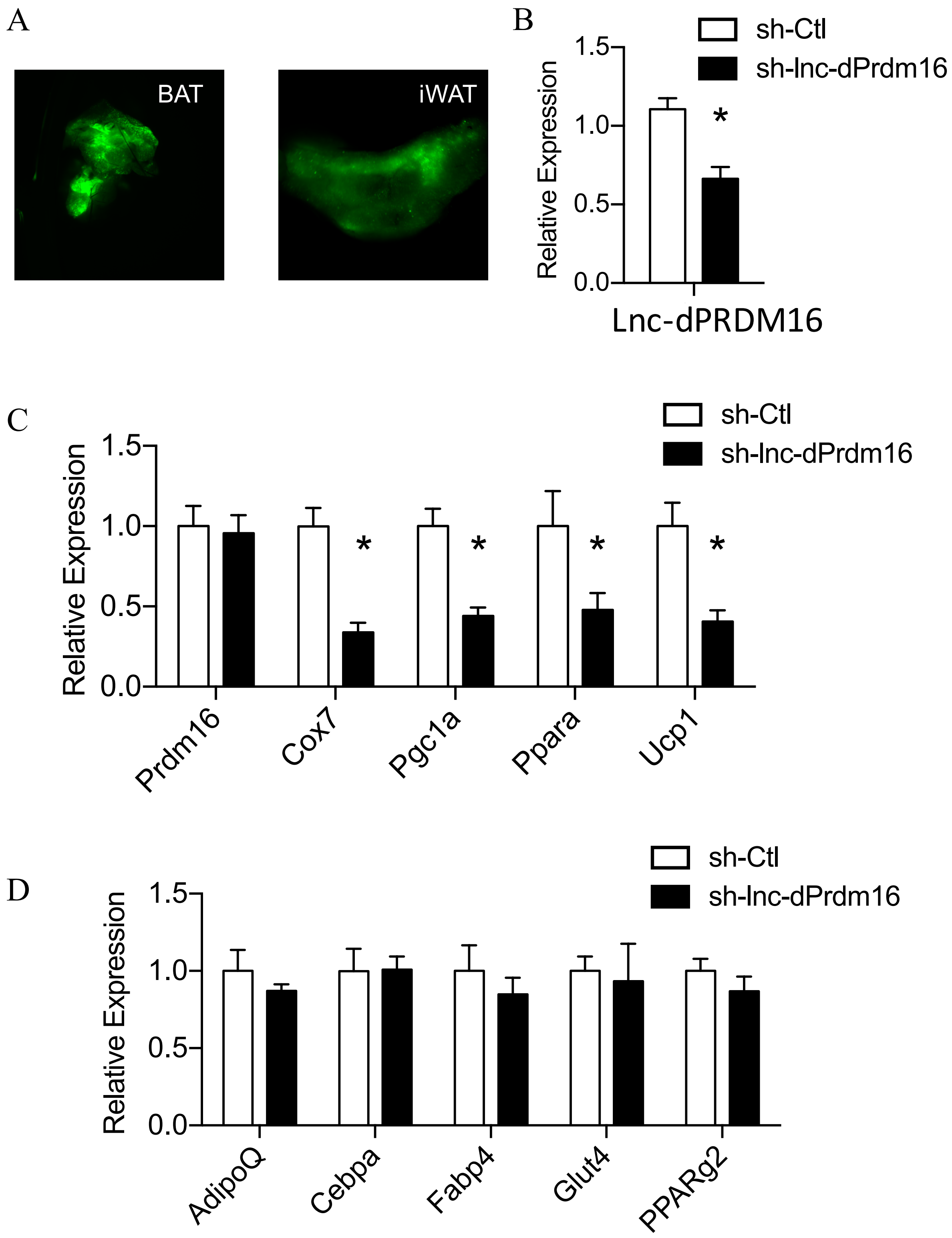

Supplementary Figure. 9 lnc-dPRDM16 is required for BAT-selective marker expression in BAT. (A) interscapular BAT and iWAT were injected by adenoviral sh-Ctl. 7 days later, the isolated BAT was observed under fluorescent microscope. Real-time PCR was performed to examine the lnc-dPRDM16(B), BAT-selective marker(C) and Pan-adipogenic markers (D) in BAT. Error bars represent mean ± SEM, n = 7, * P<0.05, Student T test.

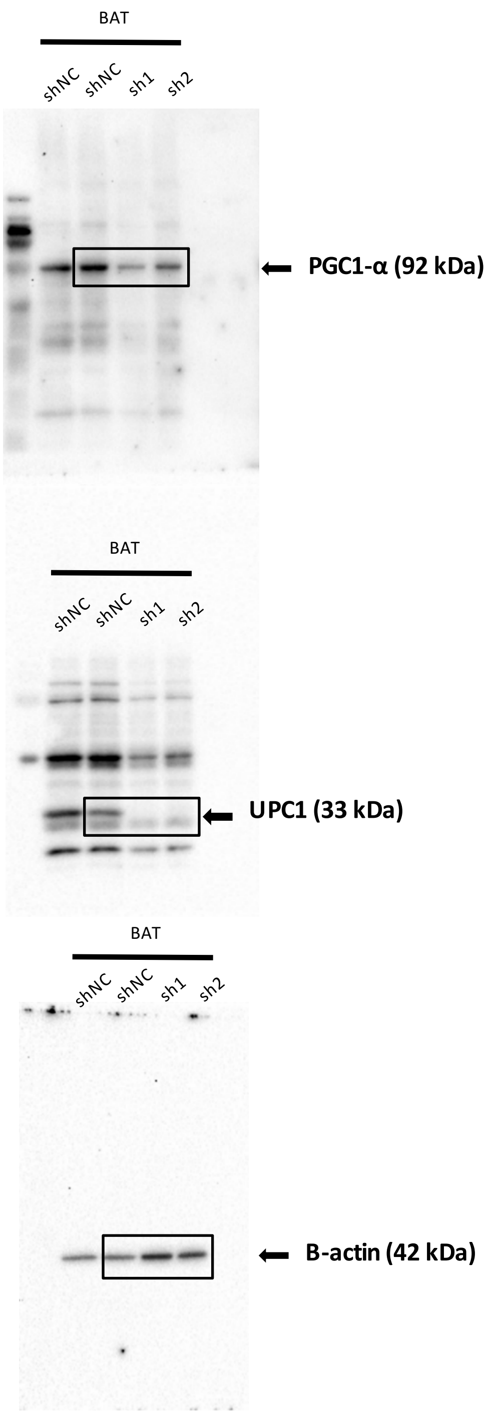

Supplementary Figure. 10. Whole gel picture of the Western blot in figure 8I

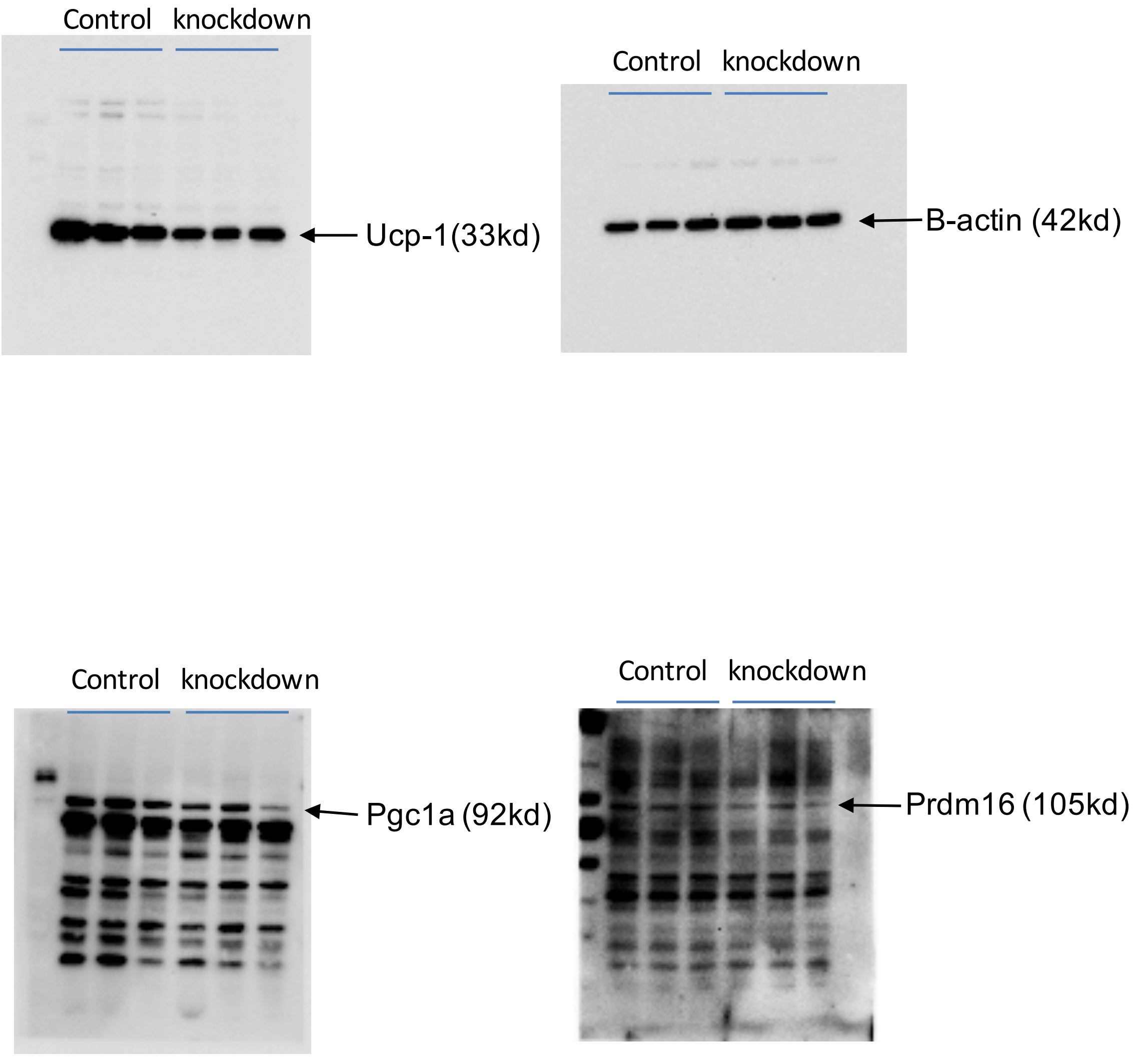

Supplementary Figure. 11. Whole gel picture of the Western blot in figure 9B.

Supplementary table1: RNA-seq dataset

Sample	Platform	Library	Number of reads	Read length	Species	Reference	Mapping rate against HG19 ensembl gene annotations (%
fiBAT1	Illumina HiSeq2000	Long polyA(+)	83,300,994	2*100	Homo sapiens	This study	89.5
fiBAT2	Illumina HiSeq2000	Long polyA(+)	87,083,828	2*100	Homo sapiens	This study	89.4
fiBAT3	Illumina HiSeq2000	Long polyA(+)	85,336,598	2*100	Homo sapiens	This study	88.5
fiBAT4	Illumina HiSeq2000	Long polyA(+)	82,007,714	2*100	Homo sapiens	This study	89.7
OME1	Illumina HiSeq2000	Long polyA(+)	66,184,956	2*100	Homo sapiens	This study	87.6
OME2	Illumina HiSeq2000	Long polyA(+)	68,701,108	2*100	Homo sapiens	This study	91.0
OME3	Illumina HiSeq2000	Long polyA(+)	60,252,096	2*100	Homo sapiens	This study	90.5
SUB1	Illumina HiSeq2000	Long polyA(+)	59,4141,900	2*100	Homo sapiens	This study	89.8
SUB2	Illumina HiSeq2000	Long polyA(+)	53,577,742	2*100	Homo sapiens	This study	87.4
SUB3	Illumina HiSeq2000	Long polyA(+)	47,306,742	2*100	Homo sapiens	This study	88.8
BAT_D0	Illumina HiSeq2000	Long polyA(+)	43,917,678	2*100	Homo sapiens	This study	91.6
BAT_D21	Illumina HiSeq2000	Long polyA(+)	40,231,502	2*100	Homo sapiens	This study	92.3
WAT_D0	Illumina HiSeq2000	Long polyA(+)	37,056,800	2*100	Homo sapiens	This study	92.6
WAT_D21	Illumina HiSeq2000	Long polyA(+)	36,388,330	2*100	Homo sapiens	This study	91.6
Pread. BAT 11-1	Ilumina HiSeq25	Total RNA	29,280,266	1*51	Homo sapien	Shinoda et al. (2015) [19]	

	00				s		
Pread. BAT 11-3	Ilumina HiSeq2500	Total RNA	29,303,433	1*51	Homo sapiens	Shinoda et al. (2015) [19]	
Pread. BAT 16	Ilumina HiSeq2500	Total RNA	30,876,838	1*51	Homo sapiens	Shinoda et al. (2015) [19]	
BAT 11-1 8	Ilumina HiSeq2500	Total RNA	35,193,845	1*51	Homo sapiens	Shinoda et al. (2015) [19]	
BAT 11-3 8	Ilumina HiSeq2500	Total RNA	32,580,055	1*51	Homo sapiens	Shinoda et al. (2015) [19]	
BAT 16 7	Ilumina HiSeq2500	Total RNA	36,461,421	1*51	Homo sapiens	Shinoda et al. (2015) [19]	
Pread. WAT 7-11	Ilumina HiSeq2500	Total RNA	30,370,572	1*51	Homo sapiens	Shinoda et al. (2015) [19]	
Pread. WAT 7-7	Ilumina HiSeq2500	Total RNA	31,606,951	1*51	Homo sapiens	Shinoda et al. (2015) [19]	
Pread. WAT 7-9	Ilumina HiSeq2500	Total RNA	38,795,258	1*51	Homo sapiens	Shinoda et al. (2015) [19]	
WAT 7-11 7	Ilumina HiSeq2500	Total RNA	32,019,056	1*51	Homo sapiens	Shinoda et al. (2015) [19]	
WAT 7-7 9	Ilumina HiSeq2500	Total RNA	35,955,050	1*51	Homo sapiens	Shinoda et al. (2015) [19]	
WAT 7-9 9	Ilumina HiSeq2500	Total RNA	32,716,306	1*51	Homo sapiens	Shinoda et al. (2015) [19]	
Sample 10 (WAT_thermoneutral)	Ilumina HiSeq2500	Total RNA	13,425,365	1*51	Homo sapiens	Chondronikola et al. (2016) [38]	76.7
Sample 7 (BAT_cold exposure)	Ilumina HiSeq2500	Total RNA	21,070,227	1*51	Homo sapiens	Chondronikola et al. (2016) [38]	73.8
Sample 8 (WAT_cold exposure)	Ilumina HiSeq2500	Total RNA	19,894,552	1*51	Homo sapiens	Chondronikola et al. (2016) [38]	74.7
Sample 9 (BAT_thermoneutral)	Ilumina HiSeq2500	Total RNA	13,010,599	1*51	Homo sapiens	Chondronikola et al. (2016) [38]	74.1

Supplementary table2: siRNA sequences

lncRNA	siRNA sequences
CUFF.43621(Snhg15)	rGrUrG rCrUrG rGrArG rCrUrA rGrArA rGrArG rArCrU rUrGA T<br>rArUrC rArArG rUrCrU rCrUrU rCrUrA rGrCrU rCrCrA rGrCrA rCrCrU
CUFF.127822.2	5' rArArArGrArArUrGrUrCrCrArCrArUrGrArArUrArCrUrGTT 3' +<br>5' rArArCrArGrUrArUrUrCrArUrGrUrGrGrArCrArUrUrCrUrUrUrUrA 3'
CUFF.140021.1	5' rCrUrGrArArGrUrGrCrUrUrUrArGrUrCrCrUrArArGrGrGTT 3'<br>5' rArArCrCrCrUrUrArGrGrArCrUrArArArGrCrArCrUrUrCrArGrCrU 3'
CUFF.163852(Snhg1)	5' rGrUrGrUrGrArArGrGrArUrArGrGrArArCrArGrArArArUCA 3'<br>5' rUrGrArUrUrUrCrUrGrUrUrCrCrUrArUrCrCrUrUrCrArCrArCrGrC 3'
CUFF.306253.1	5' rGrArGrUrCrArArCrArUrUrUrCrUrUrGrArArUrArArArUAT 3'<br>5' rArUrArUrUrUrArUrUrCrArArGrArArArUrGrUrUrGrArCrUrCrUrG 3'
CUFF.330389.5	5' rCrUrGrArUrArGrCrArUrUrGrArUrGrArArGrArArUrUrCTA 3'<br>5' rUrArGrArArUrUrCrUrUrCrArUrCrArArUrGrCrUrArUrCrArGrCrC 3'

Supplementary table3: Primer sequences

Primer name	Primer sequences	Nearby lncRNA
CUFF.43621(Snhg15)-F2	TCCACCAAAGAGATGCAGAA	
CUFF.43621(Snhg15)-R2	GCATCGGATCCCCATTACA	
CUFF.127822.2-F1	TAAGCAGGTGCCATTGGAAG	
CUFF.127822.2-R1	TGAACGTCTCTTCGTAGGGT	
CUFF.140021.1-F1	GTGTTCGTGGTCCCTTTCAT	
CUFF.140021.1-R1	ACTCAACATGCCCTGCAAAA	
CUFF.163852(Snhg1)-F1	CAAAAGGATGGGTGTACGCT	
CUFF.163852(Snhg1)-R1	AACCCACAAGTATGGCACTG	
CUFF.306253.1-F1	CCACCACAACCACACTTCAT	
CUFF.306253.1-R1	ACAGGTGTACCCAGCTACTT	
CUFF.330389.5-F1	CAGCACAAAGTCACAGGACA	
CUFF.330389.5-R1	TCCTTCCTGTGTGGACATCT	
Hmgb2-F	GCTCGTTATGACAGGGAGATG	CUFF.306253
Hmgb2-R	TTGCCCTTGGCACGGTATG	CUFF.306253
Myo1g-F	GGCCCTGAGTATGGGAAACC	CUFF.43621
Myo1g-R	GATACGAGCACCTCACCAATG	CUFF.43621
Slc3a2-F	TGATGAATGCACCCTTGTACTTG	CUFF.163852
Slc3a2-R	GCTCCCCAGTGAAAGTGGA	CUFF.163852
Rab26-F	TTGGTAGCACTGGAGACTTCT	CUFF.140021
Rab26-R	CCATCCTTGAAGCGCACAA	CUFF.140021
FAM46A-F	GAGGGCGAAGGGTACTTTGC	CUFF.330389
FAM46A-R	TTGCTCCCAGTTCAGCACATT	CUFF.330389
Masp1-F	CTTCTGTGGGGGTAGCCTTTT	CUFF.127822
Masp1-R	TGAGCTGTGTAGGGTTGGTTC	CUFF.127822
Lnc-dPRDM16-F	TCCGGTTACCCTTCTGTGAC	
Lnc-dPRDM16-R	GGGAAAATTCCTCTCCAAGC	
Prdm16-F	CAGCACGGTGAAGCCATTC	
Prdm16-R	GCGTGCATCCGCTTGTG	
Pgc1α-F	CCCTGCCATTGTTAAGACC	

Pgc1α-R	TGCTGCTGTTCCTGTTTTC	
Ucp1-F	ACTGCCACACCTCCAGTCATT	
Ucp1-R	CTTTGCCTCACTCAGGATTGG	
Cebpb-F	cgttcatgcaccgcctgctg	
Cebpb-R	aggggctgaagtcgatggcg	
Cidea-F	GCCGTGTTAAGGAATCTGCTG	
Cidea-R	TGCTCTTCTGTATCGCCCAGT	
Ppara-F	AGAGCCCCATCTGTCCTCTC	
Ppara-R	ACTGGTAGTCTGCAAAACCAAA	
lncBATE1-F1	GGACTATGAACTTTTTGGAC	
lncBATE1-R1	GTGAGATCCTCTCTCAGTGT	
RPL23-F	TGTGAAGGGAATCAAGGGAC	
RPL23-R	TGTTTACTATGACCCCTGCG	
hUCP1-F1	acacggggacttataatgcg	
hUCP1-R1	gcgcaaaatccagcgataag	
hRPL32-F	CAACATTGGTTATGGAAGCAACA	
hRPL32-R	TGACGTTGTGGACCAGGAACT	
hPRDM16 F	CGAGGCCCCTGTCTACATTC	
hPRDM16 R	GCTCCCATCCGAAGTCTGTC	
